# AI avatar tells you what happened: The first test of using AI-operated children in simulated interviews to train investigative interviewers

**DOI:** 10.3389/fpsyg.2023.1133621

**Published:** 2023-02-23

**Authors:** Shumpei Haginoya, Tatsuro Ibe, Shota Yamamoto, Naruyo Yoshimoto, Hazuki Mizushi, Pekka Santtila

**Affiliations:** ^1^Faculty of Psychology, Meiji Gakuin University, Tokyo, Japan; ^2^Independent Researcher, Tokyo, Japan; ^3^Forensic Science Laboratory, Hokkaido Prefectural Police Headquarters, Sapporo, Hokkaido, Japan; ^4^Graduate School of Medicine, University of the Ryukyus, Nishihara, Okinawa, Japan; ^5^Graduate School of Humanities and Human Sciences, Hiroshima Shudo University, Hiroshima, Japan; ^6^NYU Shanghai and NYU-ECNU Institute for Social Development, Shanghai, China

**Keywords:** child sexual abuse, investigative interviewing, simulation training, artificial intelligence, serious game

## Abstract

Previous research has shown that simulated child sexual abuse (CSA) interview training using avatars paired with feedback and modeling improves interview quality. However, to make this approach scalable, the classification of interviewer questions needs to be automated. We tested an automated question classification system for these avatar interviews while also providing automated interventions (feedback and modeling) to improve interview quality. Forty-two professionals conducted two simulated CSA interviews online and were randomly provided with no intervention, feedback, or modeling after the first interview. Feedback consisted of the outcome of the alleged case and comments on the quality of the interviewer’s questions. Modeling consisted of learning points and videos illustrating good and bad questioning methods. The total percentage of agreement in question coding between human operators and the automated classification was 72% for the main categories (recommended vs. not recommended) and 52% when 11 subcategories were considered. The intervention groups improved from first to second interview while this was not the case in the no intervention group (intervention x time: *p* = 0.007, *η_p_*^2^ = 0.28). Automated question classification worked well for classifying the interviewers’ questions allowing interventions to improve interview quality.

## 1. Introduction

Prevalence estimates for child sexual abuse (CSA) range from 8 to 31% for girls and 3 to 17% for boys (Barth et al., 2013). Given the harm caused by CSA on individuals’ psychological, relational, and somatic health (Hailes et al., 2019), it is paramount to prevent CSA but also to investigate suspected cases effectively. However, the child’s statement is often the only available evidence (Elliott and Briere, 1994; Herman, 2009) making investigative interviews with the child central. Unfortunately, while children can provide accurate reports, these can be distorted by closed and suggestive questioning (Ceci and Bruck, 1995). Unfortunately, such questions are often used (Cederborg et al., 2000; Sternberg et al., 2001; Korkman et al., 2008) and effective training strategies are needed to change this. Here, we present the results of a first training study in which interviewers interviewed AI-driven child avatars in an attempt to change their interview behavior using either a feedback or a modeling intervention between two interviews.

In order to support children’s reporting accuracy, it is important to avoid leading questions as these can lead to inaccurate statements (Ceci and Bruck, 1993, 1995; Bruck and Ceci, 1999). A study by Finnilä et al. (2003), for example, showed that after a visit at the daycare by a clown, 20% of children falsely answered yes to the question “He told you that what you did together was a secret and that you could not tell anyone, did not he?.” Instead, interviewers should use open-ended questions that promote recall memory eliciting more accurate answers from children (Lamb et al., 2003; Lyon, 2014). The challenge is to get interviewers to follow this approach to interviewing in practice (e.g., Johnson et al., 2015).

Unfortunately, most attempts at training interviewers have not been effective. An exception is the work of Michael E. Lamb and colleagues (e.g., Lamb et al., 2002b), showing that a structured interview protocol coupled with extensive feedback improves interview quality. However, performance deteriorates after feedback is no more available (Lamb et al., 2002a,b). Also, feedback on real investigative interviews is limited to the questions used but cannot be given regarding whether the elicited child responses are actually accurate or inaccurate. Another successful training program is the Specialist Vulnerable Witness Forensic Interview Training (Benson M. S. and Powell M. B., 2015; Powell et al., 2016) which includes several modules over a number of months. Using actors’ role playing children, also a commonly used approach, may not adequately mimic the cognitive abilities of real children. Finally, a practically useful approach needs to be scalable, that is, it needs to be possible to provide it effectively and at a low cost. This is not true for some of the current approaches.

In a promising approach, the efficacy of simulated avatar interview training for changing interviewer behavior has been explored by the following research groups. Powell and colleagues have developed a training tool using digital avatars that allows interviewers learn the use of open-ended questions by choosing appropriate questions from a list of options (see Brubacher et al., 2015). In this line of studies, the effectiveness of comprehensive training programs that encompass mock interviews with avatars have been examined (Benson M. and Powell M., 2015; Benson M. S. and Powell M. B., 2015; Powell et al., 2016), and more recently, long-term training effects were also examined (Brubacher et al., 2021). Baugerud and colleagues have developed interactive virtual reality avatars to train interviewers (Salehi et al., 2022). They have investigated the optimal combination of different components (e.g., visual, auditorial, emotional, and linguistic features of avatars) emphasizing avatar realism with positive feedback from potential end-users. However, evidence of efficacy has not yet been published. Volbert and colleagues have also worked on building a training module for discussions with virtual children and recently reported the algorithm of them (Tamm et al., 2022). In addition, Santtila and colleagues have developed Avatar Training using simulated avatar interviews with different interventions. This approach is characterized by a more avatar interview-centered training protocol. While Powell and colleagues include the avatar interviews as part of a training course also including other components (such as vocal exercises for open questions and watching videos demonstrating best practices), Avatar Training has focused on testing various interventions combined with simulated interviews with avatars where interviewers have asked their questions verbally. In addition, unlike the VR environment worked on by Baugerud and colleagues, Avatar Training has delivered the training *via* computers or smartphones. This allows both remote and face-to-face training. In addition, so far, Avatar Training is the only training with evidence of the changes in interviewer behavior obtained within the simulations transferring to interviews with actual children both in a mock-victim (Pompedda et al., 2020) and actual police field interview situations (Kask et al., 2022).

In the series of studies on Avatar Training, participants have been provided feedback on both the types of questions they used and the accuracy of the details they have elicited from the avatars after the interviews (Pompedda et al., 2015; Krause et al., 2017; Haginoya et al., 2020, 2021; Pompedda et al., 2020). The response algorithms determining the avatar responses have been modeled on the response behavior of actual children during interviews. For example, if the interviewer asks a question about something the avatar does not “remember,” the avatar gives no response. However if the question is repeated, there is a predetermined likelihood that the avatar will change their response to yes. This way, suggestive interviewing can elicit inaccurate details from the avatars, mimicking actual investigative interviews. The experiments conducted so far (Pompedda et al., 2015, 2020; Krause et al., 2017; Haginoya et al., 2020, 2021) have shown that this training increases use of open questions. A recent mega-analysis (Pompedda et al., 2022) including a total of 2,208 interviews containing 39,950 recommended and 36,622 non-recommended questions from 394 participants including European and Japanese students, psychologists, and police officers showed that feedback robustly increased recommended questions and decreased non-recommended questions resulting in more correct details being elicited from the avatar, and more correct conclusions being reached about what had “happened.”

Besides feedback, Haginoya et al. (2021) examined the effect of adding behavioral modeling and combining it with feedback. Behavioral modeling training (BMT) is based on Bandura and McClelland’s (1977) social learning theory and contains several components: (1) identifying well-defined behaviors, (2) showing the effective use of those behaviors through model(s), (3) giving opportunities to practice those behaviors, (4) providing feedback and social reinforcement, and (5) taking measures to maximize the transfer to actual practice (Taylor et al., 2005). Providing feedback already incorporates the last three components. Haginoya et al. (2021) incorporated the remaining components to the Avatar Training and showed that that modeling improved interview quality (and consequently also the quality of information elicited and the correctness of conclusions drawn) both alone and in combination with feedback.

The Avatar Training approach used in previous research has many positive features (e.g., the participants can communicate with the avatars in natural dialog format without having to choose from a list of options or write in their questions) and has convincing support for its efficacy, a problem remains. In all of the studies described above, an operator has coded the questions asked by the participants and input this information into the software after which the response algorithms have taken over. In order for the training approach to be truly scalable, it is necessary to automate this process, that is, the operator needs to be replaced by an automated AI classification of the questions asked. Given that the approach to classify questions developed by Lamb et al. (1996) and further developed by Korkman et al. (2006) is based on a psychological and not a linguistic analysis of interview questions, it is not immediately clear how effective such an automated classification system will be even given the impressive recent advances in natural language processing (Khurana et al., 2022). Also, investigative interviews present some particular challenges that need to be addressed by an automated classification system of interviewer questions. Importantly, the interviewer repeating a question has been proven to negatively impact the accuracy of information elicited from child witnesses (Scullin and Ceci, 2001; Krähenbühl et al., 2009; Volpini et al., 2016). This means that an automated question classifier needs to be situationally aware and identify when a question is a repetition of a previous one. Also, besides questions addressing the abuse allegation, interviewers often also ask questions about the social context of the child avatar. A failure to be able to provide appropriate responses to such questions about family relationships, hobbies, and school work would disturb the realness of the simulation. So far, no previous research has demonstrated that it is possible to use an automated question classifier and combine this with behavioral interventions to improve the quality of investigative interviews. If possible, this would constitute a major milestone in developing a scalable and effective training approach in this area.

Most research on training with avatars has so far used university student samples. However, given that professional groups may be expected to perform better given their education and experience (although experts seem to rarely use open questions in actual interviews; Lamb et al., 2018), it is important to establish training effects in these groups as well. In fact, previous research (Pompedda et al., 2020; Haginoya et al., 2021) has shown that training with avatars also improves interview quality in professional groups.

### 1.1. Aim and hypotheses

The present study had two major aims. First, we wanted to show that automated question classification is possible in the context of investigative interviews with children. Second, we wanted to show that it is possible to improve the quality of investigative interviews using behavioral interventions (feedback and modeling) while using automated question classification.

We had two hypotheses:

1. We expected it to be possible to classify interviewer questions at a higher than chance level using an automated classifier developed based on training data taken from previous experiments.
2. We expected there to be an interaction between time and intervention as a result of participants in the intervention groups improving from first to second interview while this was not expected to happen in the control group. Planned comparisons were used to investigate in which groups’ improvements occurred and which of the groups differed significantly during the second interview.

## 2. Method

### 2.1. Participants

The participants were 42 (25 women, 14 men, and three unreported gender) professionals (*M*_age_ = 37.1, *SD* = 7.1) who completed the experiment for a compensation of 2,000 JPY (13 participants were not compensated due to their employer’s regulations prohibiting this). They consisted of clinical psychologists (*n* = 16), police personnel (*n* = 8), child guidance office staff (*n* = 4), hospital workers (*n* = 4), educational facility workers (*n* = 3), and others who preferred not to specify their affiliations (*n* = 7). Twenty-four (57%) had taken training course(s) in child interviewing, and 30 (71%) had experience of interviewing children. They were randomly allocated into either the no intervention (*n* = 14), the feedback (*n* = 15) or the modeling group (*n* = 13). The Research Ethics Committee of the Faculty of Psychology of Meiji Gakuin University (Japan) approved the study (20210031) before the data collection commenced.

### 2.2. Design

The present study employed a 2 (first or second interview; within-participants) x 3 (no intervention, feedback between the two interviews, or modeling between the two interviews; between-participants) mixed design. Participants were randomly assigned to either the no intervention (*n* = 14), the feedback (*n* = 15), or the modeling (*n* = 13) group.

### 2.3. Materials

#### 2.3.1. AI-based interview simulation

**Figure 1:**
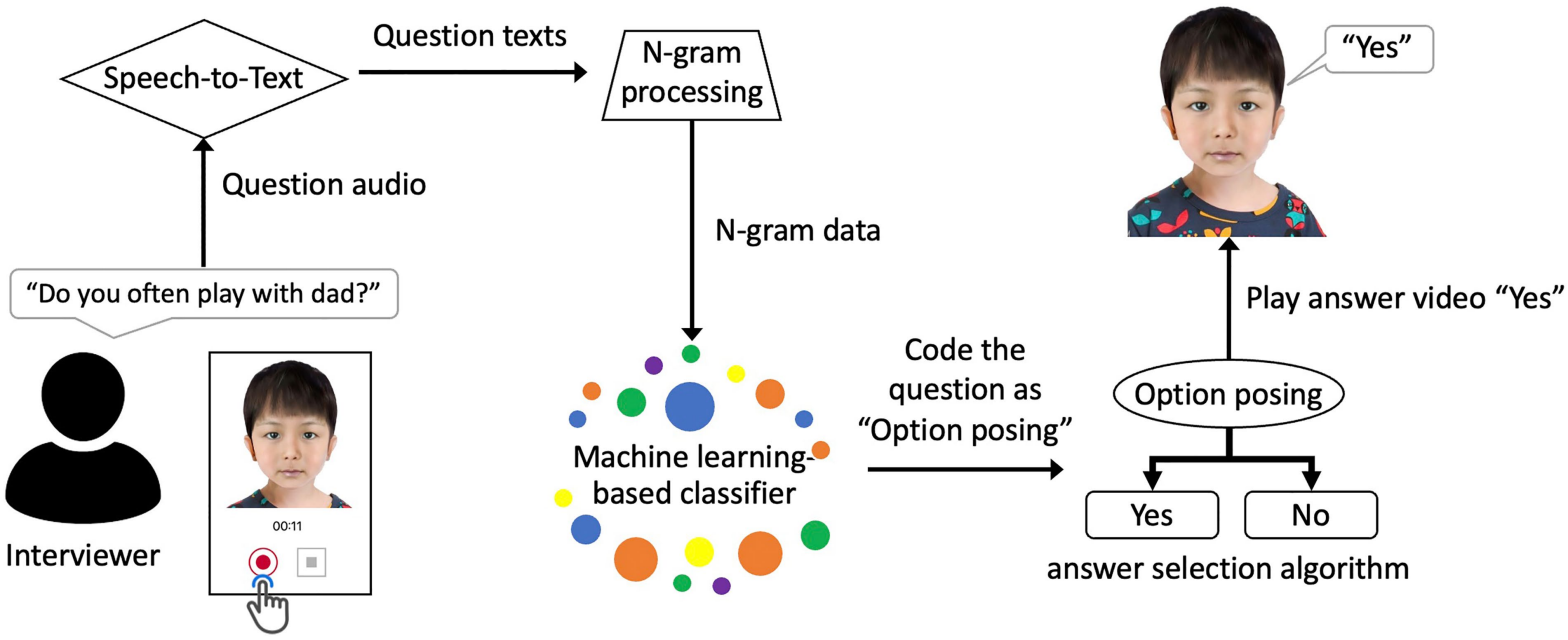
Illustration of the simulated interview process using the AI Avatar system.

To create an avatar that automatically responds to the interviewer, we developed an AI model (hereafter referred to as AI Avatar) that classifies the interviewer’s questions and selects appropriate answers without human assistance. The interview simulation application was built on the Microsoft Azure server and it was available 24/7 during the study period. Figure 1 illustrates the interview simulation (an example video is available from this link: https://youtu.be/hb9knAMzrds). The simulated interview was conducted in a question-and-answer format between the interviewer and the AI Avatar. The interviewer started recording a question by clicking the recording button presented below the avatar and ended the recording by clicking the end recording button. The recorded question was then transcribed using the Google Cloud Speech-to-Text API and sent to the N-gram extraction step. Next, the extracted N-gram was processed by a question classification algorithm based on a machine learning model and the answer selection algorithm then chose and played an avatar answer to the interviewer.

#### 2.3.2. Avatars

Of the total of 16 avatars available in the AI Avatar system, two avatars were allocated to each of the eight patterns of features with different ages (4 or 6 years old), gender (girl or boy), and abuse status (abused or non-abused). Two avatars (a 4-year old non-abused boy and a 6-year old abused girl) were used in the modeling intervention as examples of good and bad interviews and were not used in the simulated interviews.

#### 2.3.3. Question classification algorithm

The question classification algorithm of the AI Avatar categorized the transcribed questions into one of 11 possible types as described in previous studies (see Table 1; Sternberg et al., 1996; Korkman et al., 2006). The algorithm was mainly based on a tree ensemble model, XGBoost (Chen and Guestrin, 2016), which is a machine learning technique. The XGBoost model was used in the present research as it has been recognized as an accurate classifier and requires minimal tuning (Shwartz-Ziv and Armon, 2022). The XGBoost model was developed using 8,754 questions asked in Japanese and their classification results by human operators collected in previous research (Haginoya et al., 2020, 2021, Unpublished results^1^) as training data. The XGBoost model builds a classification model by creating new models from previous models’ residuals and combines them to make the final prediction. This method was used to process a large number of variables generated from N-gram patterns. The N-gram patterns of the questions were used as the predictors for question classification in the XGBoost model. N-gram is a set of N adjacent characters, words, or phrases. For example, if the recommended question “Tell me what happened” is processed by bigrams of words, three variables “tell me,” “me what,” and “what happened” are obtained, and the value of “1″ is input for each as their frequency. Regarding the training data in the present study, N was set from 1 to 5, and N-grams were extracted in character units as this is the appropriate approach for Japanese. Each extracted N-gram was treated as a variable, and the XGBoost model performed question classification based on the frequency of N-grams calculated for each question. The hyperparameters of the trained model were number of estimators (200), max depth (3), min child weight (100), subsample (0.9), colsample bytree (0.9), reg lambda (0.05), reg alpha (0.05), and learning rate (0.01). All parameters except learning rate were tuned using cross-validation scores with a 5-fold splitting of the training data. The SMOTETomek resampling technique (Batista et al., 2004) was employed to balance the unequal distribution of classes in the training data. The data were allocated into training data (80%) and test data (20%) using stratified splitting (see Supplementary material 1 for class distributions of training and test data). The classification accuracy of the test data with the developed model was 71%.

**Table 1:** Description and examples by question types.

Question types	Description	Examples
*Recommended questions*		
Invitation broad	Open-ended and non-suggestive questions that elicit free narrative from children.	“Tell me everything that happened from the beginning to the end”
Invitation focus	Open-ended and non-suggestive questions that elicit narrative about the focused topic from children.	“Tell me about your family”
Facilitators	Non-suggestive questions that promote further narrative about the content previously mentioned.	“Continue” “Go ahead” “Ok”
Directive	Questions that focus the children’s attention on the content the child has already mentioned for further explanation.	“Where did you go with your mom?” “What game did you play?”
Clarification	Attempts to clarify what the child has said.	“What did you say?” “I did not hear you well, so tell me again”
*Not recommended questions*		
Option posing	Closed questions that focus the children’s attention on content that the child had not yet mentioned without implying a specific type of answer.	“Do you play with dad?”
Specific suggestive	Questions that indicate what kind of answer is expected by assuming details that children have not mentioned.	“Did your dad do something bad to you?” “Is your dad a bad person?”
Unspecific suggestive	Questions that indicate what kind of answer is expected without assuming details that children have not mentioned.	“I know that you have something to talk about, tell me!”
Repetition	Questions continuously asking what the interviewer has already asked.	
Inappropriate utterance for children	Questions that are not appropriate to elicit correct information from children such as questions asking more than one detail at once, containing difficult words for children, are grammatically unclear, relying on temporal cognitive processes, and encouraging children’s fantasies.	“Where were you with your father, what were you doing after that?” “What is the relationship between mom and dad?” “When did your mom leave the park?” “If you were your dad, what would you do?”
Multiple-choice	Questions that focus the children’s attention on specific answers, or force them to choose among options.	“Did you go practicing football with Kanta or Miura?”

In addition to machine learning, a series of additional rules were implemented in the question classification algorithm to improve the accuracy of question classification. For example, questions that could be classified with high accuracy by a specific character pattern, such as a greeting (e.g., “Hello,” “How old are you”) at the beginning of an interview were processed by a specifically set if-then rule instead of a machine learning model. In addition, there are questions that should have different classification results depending on keywords that suggest abusive experience even if the question otherwise resembles a recommended question type. For example, the question “Tell me about someone hurting you” should be classified as suggestive-open rather than as an invitation-focus question in case the child has not mentioned anything about a painful experience. To obtain an accurate classification for these questions, a list of suggestive keywords was created based on the training data and was used to determine the presence of suggestive keywords in a question. Furthermore, to detect the repetition of questions, a Jaccard coefficient was calculated as the similarity of the N-gram patterns between consecutive questions for determining whether a question was a repetition of the immediately preceding question. The relationship between the two questions can be represented by a 2×2 contingency table. The Jaccard coefficient is a measure of similarity that does not consider cases where attributes (N-gram patterns in the present study) were not present in either of the two sample sets (questions in the present study) as similarity.

This characteristic of the Jaccard coefficient fits the data structure of the present study, in which the frequency of each N-gram pattern is 0 for most of the questions contained in the training data. The threshold value of the Jaccard coefficients was set considering the classification accuracy of the repetition of questions in the training data. In addition, to make the avatar’s responses resemble those of a real child, topic keywords (e.g., “Dad”) corresponding to predefined responses (e.g., “Daddy is kind to me,” “Daddy never tells me fairytales,” “Sometimes daddy plays with me”) of each avatar were tagged on the input question (e.g., “Tell me about your dad”) when contained in a recommended question. AI Avatar had lists of avatar responses with their presentation order number corresponding to each keyword tag. An avatar response with the lowest number among unplayed ones on the response list of a tagged keyword was played to give the interviewer an answer that met the context of the question.

#### 2.3.4. Answer selection algorithm

The answer selection algorithm implemented in the AI Avatar adopted the method used in previous research on Avatar Training (Pompedda et al., 2015, 2017, 2020; Krause et al., 2017; Haginoya et al., 2020, 2021). In this method, a set of avatar responses with predefined selection probabilities following each question type. Three types of answers were possible: Answers related to the alleged abuse case (hereinafter referred to as relevant details), answers unrelated to the alleged abuse case (hereinafter referred to as neutral details), and incorrect details (information that contradicts details held in the avatar’s “memory”). Relevant and neutral details are only provided as responses to recommended questions. When the interviewer asked a recommended question, the probability of eliciting relevant or neutral details was 20% each time for 4 years old avatars and 25% each time for 6 years old avatars. The difference in probabilities between ages derives from previous research on the informativeness of children’s answers to recommended questions (Malloy et al., 2017).

Each avatar had nine relevant details in their memory, and these were provided one at a time. The relevant details were provided in a fixed order with the last four containing the information necessary to reach the correct conclusion about both abused and non-abused cases. Therefore, the difficulty of reaching the correct conclusion was comparable between abused and non-abused cases. Incorrect details (details that contradicted the information held by the avatar) could be created by the interviewer by asking not-recommended questions. The information held by the avatar is predefined as a set of responses, and inconsistencies with them were defined as erroneous information. For example, if the interviewer asked an avatar that does not have information about any abusive experience “Did your dad hurt you?,” and the response algorithm returns “Yes,” an incorrect detail has been created. The answer selection algorithms were developed based on findings of experimental research on children, analyses of records of child interviews in legal settings, and theoretical research on children’s memory. Previous research using these algorithms (Pompedda et al., 2015, 2017, 2020; Krause et al., 2017; Haginoya et al., 2020, 2021) have shown a positive correlation with the number of relevant details and a negative correlation with the number of incorrect details concerning the percentage of recommended questions, a pattern observed in interviews with real children, thereby supporting the ecological validity of the answer selection.

### 2.4. Procedure

The mean length of experimental sessions was 52.1 min (SD = 14.4). After completing the informed consent and demographic information forms, the participants read short instructions (see Supplementary material 2) on best practice in interviewing children and answered two questions to confirm their understanding of these instructions. Participants were asked to read the instructions again if they gave an incorrect answer to one or both of the two questions.

Participants conducted simulated interviews with randomly selected two avatars out of the 14 available. Before each of the interviews, the participants first read a background scenario (see Supplementary material 3 for an example) of the alleged case and answered two questions about their preliminary impression of the case before the interview: (1) the presence of abuse (“present” or “absent”) and (2) confidence in their assessment on a 6 point scale (“50%: guessing” to “100%: completely sure”). Participants were instructed to focus their questions on eliciting information to determine the presence or absence of sexual abuse. Otherwise, they were free to ask any questions without restrictions. Each interview lasted 10 min.

After the interview, participants were asked three questions about their conclusions based on the information obtained in the interview: (1) the presence of the abuse (“present” or “absent”), (2) confidence in their assessment on a 6 point scale (“50%: guessing” to “100%: completely sure”), and (3) a description of what, according to them, had happened to the avatar. Unfortunately, the answers to these questions were missing for 27 (64%) participants due to a system error. Therefore, the correctness of conclusions was not included in the statistical analyses.

Between the two interviews, participants received either no intervention or either feedback or modeling as a training intervention. As the feedback intervention, the participants were provided two types of feedback after the first interview: (1) feedback consisting of the outcome of the case and (2) feedback on the questions (two recommended questions and two not recommended questions) asked in the interview. For feedback concerning questions, the AI avatar chose questions randomly from the questions recorded during the interview and then provided automated feedback regarding them. The modeling intervention included (1) reading a series of learning points of good and bad questioning methods and (2) watching a total of four 2.5-min videos of good and bad interviews with both an abused and a non-abused avatar. The contents of the modeling intervention were the same as those in Haginoya et al. (2021). Participants read the background scenarios leading to the alleged cases before watching the modeling videos of each avatar and read the outcomes of the cases after watching the modeling videos.

### 2.5. Statistical analyses

The fourth and fifth authors of this paper recoded 48% (40 interviews by 20 participants, 1,532 questions) of completed interviews in the present research. They were also involved in coding questions in all three data sets (Haginoya et al., 2020, 2021, see footnote 1) for training the question classification model. The total percentage of agreement in coding between the authors and the AI Avatar and Cohen’s kappa (κ) were calculated for 11 question types and their two categories (recommended and not recommended questions) each.

A 2 (time: first to second; within-participants) x 3 (intervention: no intervention, feedback, and modeling; between-participants) mixed design two-way MANOVA was performed on the number of recommended questions, the number of not recommended questions, the proportion of recommended questions, number of relevant details, and number of incorrect details. Planned comparisons were conducted to identify where exactly the significant differences appeared.

## 3. Results

### 3.1. Question classification accuracy

The total percentage of agreement in coding the 11 question types between operators and the AI Avatar was 52% (chance level = 8%), with a Cohen’s kappa (κ) of.42, *p* < 0.001, 95% CI [0.38, 0.45]. This can be considered moderate agreement (Landis and Koch, 1977).

The total percentage of agreement in coding the two main categories (recommended vs. not recommended) of questions between operators and the AI Avatar was 72% (chance level = 33%), with a Cohen’s kappa (κ) of.49, *p* < 0.001, 95% CI [0.45, 0.53]. This can also be considered moderate agreement (Landis and Koch, 1977). These results support our first hypothesis.

### 3.2. Descriptive statistics of question types and details

For the first interview (before the interventions), the overall means were 9.8 (SD = 5.4) for the number of recommended questions, 19.9 (SD = 8.1) for the number of not recommended questions, 34.4 (SD = 17.6) for the proportion of recommended questions, 1.2 (SD = 1.5) for the number of relevant details, and 5.2 (SD = 2.8) for the number of incorrect details.

### 3.3. Correlations between question types and details

Table 2 shows the correlations between the dependent variables. The number of recommended questions had a significant positive correlation with the number of relevant details, whereas the number of not recommended questions had a significant positive correlation with the number of incorrect details. The proportion of recommended questions had a significant positive correlation with the number of relevant details, and a significant negative correlation with the number of incorrect details. Therefore, the algorithms worked as expected. The numbers of relevant and incorrect details were unrelated, *r* = −0.08, *p* = 0.482.

**Table 2:** Correlations between question types and details.

Variables	Relevant details	Incorrect details
Number of recommended questions	0.63***	−0.11
Number of not recommended questions	−0.21	0.64***
Recommended questions (%)	0.51***	−0.39***

***p < 0.001.

### 3.4. Multivariate and univariate level significant effects and planned comparisons

Significant effects on the combined dependent variables were found for time, *F* (5, 35) = 9.90, *p* < 0.001, *η_p_*^2^ = 0.59, 1−*β* = 1.00, and for the interaction between intervention and time, *F* (10, 70) = 2.72, *p* = 0.007, *η_p_*^2^ = 0.28, 1−*β* = 0.95, while there was no significant main effect for intervention, *F* (10, 70) = 1.01, *p* = 0.444, *η_p_*^2^ = 0.13, 1−*β* = 0.49. The significant interaction supports our second hypothesis.

Univariate level significant effects of time emerged for the number of recommended questions, *F* (1, 39) = 46.95, *p* < 0.001, *η_p_*^2^ = 0.55, 1−*β* = 1.00, the proportion of recommended questions, *F* (1, 39) = 24.45, *p* < 0.001, *η_p_*^2^ = 0.39, 1−*β* = 1.00, and the number of relevant details, *F* (1, 39) = 6.65, *p* = 0.014, *η_p_*^2^ = 0.15, 1−*β* = 0.71, but not for the number of not recommended questions, *F* (1, 39) = 2.36, *p* = 0.132, *η_p_*^2^ = 0.06, 1−*β* = 0.32, and the number of incorrect details, *F* (1, 39) = 0.51, *p* = 0.479, *η_p_*^2^ = 0.01, 1−*β* = 0.11.

At a univariate level, significant effect of intervention emerged for the number of relevant details, *F* (2, 39) = 3.50, *p* = 0.040, *η_p_*^2^ = 0.15, 1−*β* = 0.62, but not for the number of recommended questions, *F* (2, 39) = 1.38, *p* = 0.264, *η_p_*^2^ = 0.07, 1−*β* = 0.28, the number of not recommended questions, *F* (2, 39) = 0.40, *p* = 0.674, *η_p_*^2^ = 0.02, 1−*β* = 0.11, the proportion of recommended questions, *F* (2, 39) = 0.71, *p* = 0.499, *η_p_*^2^ = 0.04, 1−*β* = 0.16, and the number of incorrect details, *F* (2, 39) = 0.30, *p* = 0.742, *η_p_*^2^ = 0.02, 1−*β* = 0.09.

Univariate level significant interaction between time and intervention emerged for the number of recommended questions, *F* (2, 39) = 9.62, *p* < 0.001, *η_p_*^2^ = 0.33, 1−*β* = 0.97, the number of not recommended questions, *F* (2, 39) = 12.25, *p* < 0.001, *η_p_*^2^ = 0.39, 1−*β* = 0.99, the proportion of recommended questions, *F* (2, 39) = 12.10, *p* < 0.001, *η_p_*^2^ = 0.38, 1−*β* = 0.99, and the number of incorrect details, *F* (2, 39) = 3.97, *p* = 0.027, *η_p_*^2^ = 0.17, 1−*β* = 0.68, but not for the number of relevant details, *F* (2, 39) = 1.43, *p* = 0.251, *η_p_*^2^ = 0.07, 1−*β* = 0.29. These interactions partly support our second hypothesis.

**Figure 2:**
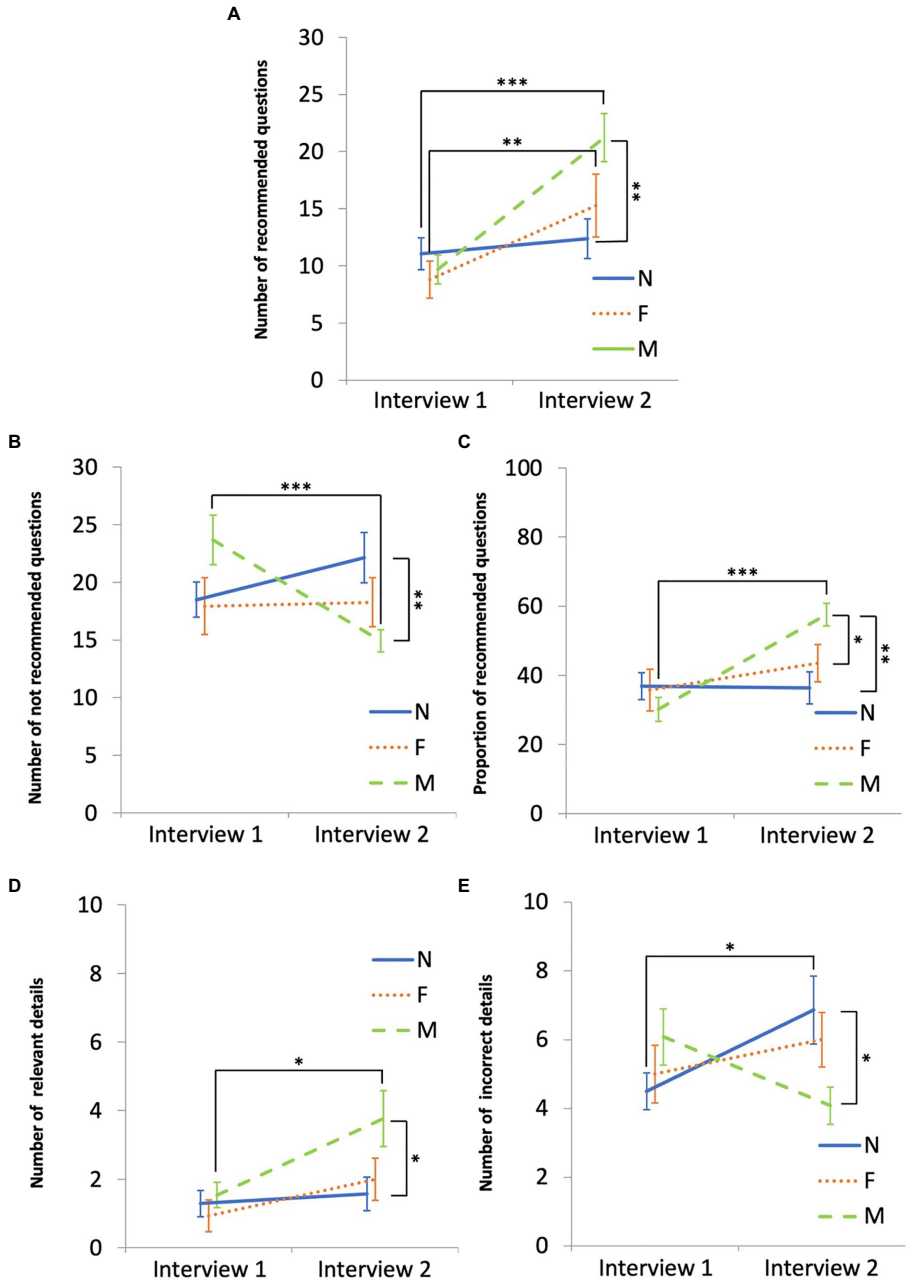
Interview quality and elicited details in the no intervention (N), feedback (F), and modeling (M) groups. In panels **(A–E)**, the x-axis indicates the first and the second interview. Panels **(A–C)** display the use of recommended and not recommended questions by the groups. Panels **(D,E)** display the elicited details (relevant and incorrect) by the groups. The error bars represent standard errors. *p < 0.05, **p < 0.01, ***p < 0.001.

Figure 2 shows the significant statistical differences in the planned comparisons between the first and second interviews within each group. The no-intervention group elicited a significantly greater number of incorrect details in the second interview (vs. the first). The feedback group used a significantly greater number of recommended questions in the second interview (vs. the first). The modeling group used a significantly greater number and proportion of recommended questions and a significantly smaller number of not recommended questions, and elicited a significantly greater number of relevant details in the second interview (vs. the first).

Significant statistical differences in the planned comparisons between groups for the first and second interviews are also shown in Figure 2. The modeling group used a significantly greater number and proportion of recommended questions and a significantly smaller number of not recommended questions compared to the no intervention group at the second interview. The modeling group also used a significantly greater proportion of recommended questions compared to the feedback group at the second interview. These results partly support our second hypothesis. There were no differences between the groups at the first interview, showing that the randomization of the participants into the three groups had been successful.

## 4. Discussion

### 4.1. Accuracy of automated question classification

The present study examined the accuracy of question classification by AI and the effectiveness of CSA interview simulations using AI-based question classification combined with two interventions (feedback and modeling) shown to be effective in previous research. Regarding the question classification, higher-than-chance-level classification accuracy was obtained both for 11 question types and for their two supra-categories (recommended and not recommended), indicating that AI worked well for classifying the interviewers’ questions. Question classification accuracy is an important factor in mimicking the experience of interviewing a real child, where recommended questions elicit informative utterances from a child, while not recommended questions elicit shorter responses sometimes even containing erroneous details. In the AI Avatar interviews, randomly chosen questions were used for the feedback intervention. Therefore, the accuracy of question classification was also required to provide accurate feedback. Importantly, the number of recommended questions significantly increased in the feedback group, suggesting that feedback improved participants’ questioning skills. Given that the discrimination between recommended and not recommended questions is essential for feedback to be effective in enhancing use of recommended questions, the classification accuracy of over 70% for these two categories is an important result of the present study.

### 4.2. Improvements in the quality of investigative interviews using interventions

Importantly, the simulated interviews with AI Avatars could be used to improve interviewing skills and quality of the information elicited from the avatars when combined with feedback and modeling interventions. The increase in the number of recommended questions shown in the feedback group has also been demonstrated robustly in a mega-analysis (Pompedda et al., 2022) that combined nine studies and examined the effects of feedback in Avatar Training. We were able to show that this feedback effect emerged even in an environment where question classification and feedback were completely automated.

Modeling resulted in more robust improvements than feedback, including a decrease of not recommended questions and increase of relevant details, with many significant differences compared to the no intervention group. These improvements are consistent with a previous study that examined modeling (Haginoya et al., 2021), which showed that modeling was as effective in interview simulations with AI Avatars as in operator-led training. This is perhaps not particularly surprising given that the delivery of the modeling intervention is not similarly dependent on question classification as feedback is. Of course, less than perfect classification of question types would reduce any intervention effect.

The difference in training effectiveness between feedback and modeling (i.e., the latter being more effective than the former) found here is also consistent with Haginoya et al. (2021). Modeling has resulted in robust improvements even if it is provided fewer times compared to feedback, where improvement requires multiple interventions over a series of interviews (Haginoya et al., 2021). The fact that only two interviews were conducted with feedback provided only once may explain the difference between the feedback intervention, which was unlikely to have reached its maximum impact, and the modeling intervention, for which a large improvement effect could be expected with a single intervention. On the other hand, given that a decrease in not recommended questions was shown by the mega-analysis already after it having been provided once (Pompedda et al., 2022), additional factors may be involved in the lack of a feedback effect for this variable in the present study. A possible explanation is the observer’s influence (also known as the Hawthorne effect) on the participants’ behavior during the interview simulation. Extensive research has been conducted on observer influence (McCambridge et al., 2014). For example, a study examining the hand sanitization behavior of health care workers showed that participants’ behavior changed when a human observer directly observed their behavior (e.g., Hagel et al., 2015), while the electronic dispenser devices that automatically count hand sanitization events show little impact (Boyce, 2017). In previous research on Avatar Training, participants interviewed avatars while being aware of the operator’s presence either in person (e.g., Krause et al., 2017; Pompedda et al., 2017) or *via* voice call (Haginoya et al., 2020, 2021) in the simulated interviews. Therefore, the training without an operator might result in a lack of observer influence and might cause a difference in the participants’ learning behavior compared to previous research. However, given that similar training effects were shown in the modeling condition as in previous research (Haginoya et al., 2021), additional factors may be involved.

Another potential explanation is the form of feedback, which differed from previous research. All previous research on Avatar Training provided feedback on recommended and not recommended questions chosen by a human operator verbally and *via* texts. However, in the current study, the AI Avatar chose the questions and provided feedback in text format. A systematic review of the effects of online learning from automated feedback (Cavalcanti et al., 2021) found that automated feedback improved learner performance in 65% of the studies with no evidence that manual feedback is more efficient than automated feedback in 83% of the studies. Therefore, if there is no critical difference in the content and quality of feedback between manual and automated feedback, the effect of feedback format on learning effectiveness may not be noteworthy. However, the total percentage of agreement between the AI Avatar and human operators in coding questions (11 question types: 52%; desirable and undesirable questions: 72%) was lower than that between operators in previous research (e.g., Haginoya et al., 2020: 74%; Haginoya et al., 2021: 80%), suggesting that the classification accuracy of the questions used for feedback may have been lower. Since using misclassified questions in feedback undermines learning optimal questioning skills, highly accurate question classification is needed to avoid this potential risk.

In addition, the present research recruited a sample of professionals, including clinical psychologists and police personnel. The results showed a level of training effectiveness similar to previous research on professionals (Pompedda et al., 2020; Haginoya et al., 2021; Kask et al., 2022) and suggest that AI Avatars are useful even for professional groups. It should also be noted that previous research has shown improvements in interviewing skills after the third interview out of 4–6 simulated interviews. Thus, the improvement in the second interview shown in the present research is particularly impressive.

### 4.3. Limitations and future implications

The present research is the first paper to demonstrate the effectiveness of investigative interviewer training using AI-driven avatars, showing the potential for scalable training. However, the results have some limitations.

Regarding the question classification using the AI Avatar, the results showed significantly higher accuracy than the chance level. However, as noted above, the total percentage of agreement between the AI Avatar and human operators was lower than that between operators in previous research. Although part of the disagreement can be assumed to be due to misclassification by operators as the question type categories are complex, the main reason for the low agreement can be attributed to the less than perfect classification accuracy of AI Avatar. As mentioned above, question classification accuracy may affect the effectiveness of feedback. Given that low classification accuracy can affect the learner’s experience by giving irrelevant and wrong feedback (e.g., providing positive feedback on a not recommended question and negative feedback on a recommended question), future improvement is desirable. As a potential approach to improve the question classification accuracy, examining more advanced models such as the Bidirectional Encoder Representations from Transformers (BERT; Devlin et al., 2018) and comparisons among multiple models should be the subject of future research.

It should also be noted that only the results of a single interview (i.e., the second interview) could show intervention effects. This may not have been a problem for the modeling intervention as previous research supports the efficacy of a single administration (Haginoya et al., 2021). However, feedback has been repeatedly reported to improve interviewing skills gradually over a number of interviews (e.g., Krause et al., 2017; Haginoya et al., 2020; Kask et al., 2022). Therefore, future research should employ a larger number of interviews, which will allow for examining a trend in the effect of feedback over time in interview simulations with AI Avatars.

Although the present research showed robust improvements using modeling, a need to test the transfer of this learning effect is necessary separately for modeling interventions. As described above, the interviewer was free to ask questions without restrictions and avatar responses were chosen from a set of responses with predefined selection probabilities following each question type. This environment, which provides a variety of contexts, encourages interviewers to customize the questions learned from the model flexibly and may develop questioning skills that can be applied to interviews with real children. Given that several factors (e.g., presenting both negative and positive models, instructing trainees to set goals, and training also trainees’ superiors) were pointed out to maximize transfer regarding behavioral modeling (Taylor et al., 2005), the transfer of interviewing skills using modeling is a promising approach to be tested in future research.

Unfortunately, we could not use conclusion correctness in our analyses. However, this is less of a problem than it may seem given that a correct conclusion is made possible by uncovering the memories of the avatar while not eliciting incorrect information from it. These latter variables were measured and shown to be impacted by the interventions.

Moreover, automated and scalable training needs to be investigated using a larger sample. Previous research on interview training has generally only used up to a hundred participants in total due to the need to conduct individual training sessions for each participant. However, the automated training protocol used in the present research can train several participants in parallel by adjusting the available number of accesses and processing capacity of the server accessed by the participants. To provide potential trainees with more training opportunities regardless of time and place, future research is needed to further improve the AI Avatars.

## Data availability statement

The raw data supporting the conclusions of this article will be made available by the authors, without undue reservation.

## Ethics statement

The studies involving human participants were reviewed and approved by the Research Ethics Committee of the Faculty of Psychology of Meiji Gakuin University. Written informed consent for participation was not required for this study in accordance with the national legislation and the institutional requirements.

## Author contributions

SH and PS contributed to the conception, design of the study, and wrote the first draft of the manuscript. SH and TI collected the data. SH performed the statistical analyses. All authors contributed to the interpretation of the analyses, revision of the manuscript, edited and gave final approval for publication, and were accountable for this work.

## Funding

This research was supported by grants from HAYAO NAKAYAMA Foundation for Science & Technology and Culture (grant number: R1-A1-29) and the NYU Shanghai Start Up Fund.

## Conflict of interest

The authors declare that the research was conducted in the absence of any commercial or financial relationships that could be construed as a potential conflict of interest.

## Publisher’s note

All claims expressed in this article are solely those of the authors and do not necessarily represent those of their affiliated organizations, or those of the publisher, the editors and the reviewers. Any product that may be evaluated in this article, or claim that may be made by its manufacturer, is not guaranteed or endorsed by the publisher.

## Footnotes

^1^Haginoya, S., Yamamoto, S., Mizushi, H., Yoshimoto, N., and Santtila, P. (unpublished results) Improving supportiveness and questioning skills using online simulated child sexual abuse interviews with feedback. [unpublished manuscript]

## References

[ref1] Bandura A. McClelland D. C. (1977). Social Learning Theory. Englewood Cliffs. Hoboken, NJ: Prentice-Hall.

[ref2] Barth J. Bermetz L. Heim E. Trelle S. Tonia T. (2013). The current prevalence of child sexual abuse worldwide: a systematic review and meta-analysis. Int. J. Public Health 58, 469–483. doi: 10.1007/s00038-012-0426-1, PMID: 23178922

[ref3] Batista G. E. A. P. A. Prati R. C. Monard M. C. (2004). A study of the behavior of several methods for balancing machine learning training data. ACM SIGKDD Explor. Newsl. 6, 20–29. doi: 10.1145/1007730.1007735

[ref4] Benson M. Powell M. (2015). Organisational challenges to delivering child investigative interviewer training via e-learning. Int. J. Police Sci. Manag. 17, 63–73. doi: 10.1177/1461355715580912

[ref5] Benson M. S. Powell M. B. (2015). Evaluation of a comprehensive interactive training system for investigative interviewers of children. Psychol. Public Policy Law 21, 309–322. doi: 10.1037/law0000052

[ref6] Boyce J. M. (2017). Electronic monitoring in combination with direct observation as a means to significantly improve hand hygiene compliance. Am. J. Infect. Control 45, 528–535. doi: 10.1016/j.ajic.2016.11.029, PMID: 28456322

[ref7] Brubacher S. P. Powell M. Skouteris H. Guadagno B. (2015). The effects of e-simulation interview training on teachers' use of open-ended questions. Child Abuse Negl. 43, 95–103. doi: 10.1016/j.chiabu.2015.02.004, PMID: 25703802

[ref8] Brubacher S. P. Shulman E. P. Bearman M. J. Powell M. B. (2021). Teaching child investigative interviewing skills: long-term retention requires cumulative training. Psychol. Public Policy Law 28, 123–136. doi: 10.1037/law0000332

[ref9] Bruck M. Ceci S. J. (1999). The suggestibility of children’s memory. Annu. Rev. Psychol. 50, 419–439. doi: 10.1146/annurev.psych.50.1.419 10074684

[ref10] Cavalcanti A. P. Barbosa A. Carvalho R. Freitas F. Tsai Y. S. Gašević D.. (2021). Automatic feedback in online learning environments: a systematic literature review. Comp. Educ. Artif. Intell. 2:100027. doi: 10.1016/j.caeai.2021.100027

[ref11] Ceci S. J. Bruck M. (1993). Suggestibility of the child witness: a historical review and synthesis. Psychol. Bull. 113, 403–439. doi: 10.1037/0033-2909.113.3.403, PMID: 8316609

[ref12] Ceci S. J. Bruck M. (1995). Jeopardy in the Courtroom. A scientific analysis of children’s testimony. Washington, DC: American Psychological Association.

[ref13] Cederborg A. C. Orbach Y. Sternberg K. J. Lamb M. E. (2000). Investigative interviews of child witnesses in Sweden. Child Abuse Negl. 24, 1355–1361. doi: 10.1016/S0145-2134(00)00183-6, PMID: 11075701

[ref14] Chen T. Guestrin C. (2016). “XGBoost: a scalable tree boosting system” in Proceedings of the ACM SIGKDD International Conference on Knowledge Discovery and Data Mining, 13-17-August-2016, 785–794.

[ref16] Devlin J. Chang M. W. Lee K. Toutanova K. (2018). Bert: pre-training of deep bidirectional transformers for language understanding. arXiv preprint arXiv:1810.04805. doi: 10.48550/arXiv.1810.04805

[ref17] Elliott D. M. Briere J. (1994). Forensic sexual abuse evaluations of older children: disclosures and symptomatology. Behav. Sci. Law 12, 261–277. doi: 10.1002/bsl.2370120306

[ref18] Finnilä K. Mahlberg N. Santtila P. Sandnabba K. Niemi P. (2003). Validity of a test of children’s suggestibility for predicting responses to two interview situations differing in their degree of suggestiveness. J. Exp. Child Psychol. 85, 32–49. doi: 10.1016/S0022-0965(03)00025-0, PMID: 12742761

[ref19] Hagel S. Reischke J. Kesselmeier M. Winning J. Gastmeier P. Brunkhorst F. M.. (2015). Quantifying the Hawthorne effect in hand hygiene compliance through comparing direct observation with automated hand hygiene monitoring. Infect. Control Hosp. Epidemiol. 36, 957–962. doi: 10.1017/ice.2015.93, PMID: 25903555

[ref20] Haginoya S. Ibe T. Yamamoto S. Yoshimoto N. Mizushi H. Santtila P. (2022). AI avatar tells you what happened: the first test of using AI-operated children in simulated interviews to train investigative interviewers. PsyArXiv [Preprint]. doi: 10.31234/osf.io/pyqxb, (Accessed December 29, 2022) PMC9995382 36910814

[ref22] Haginoya S. Yamamoto S. Pompedda F. Naka M. Antfolk J. Santtila P. (2020). Online simulation training of child sexual abuse interviews with feedback improves interview quality in Japanese university students. Front. Psychol. 11:998. doi: 10.3389/fpsyg.2020.00998, PMID: 32528374 PMC7265454

[ref23] Haginoya S. Yamamoto S. Santtila P. (2021). The combination of feedback and modeling in online simulation training of child sexual abuse interviews improves interview quality in clinical psychologists. Child Abuse Negl. 115:105013. doi: 10.1016/j.chiabu.2021.105013, PMID: 33639559

[ref24] Hailes H. P. Yu R. Danese A. Fazel S. (2019). Long-term outcomes of childhood sexual abuse: an umbrella review. Lancet Psychiatry 6, 830–839. doi: 10.1016/S2215-0366(19)30286-X, PMID: 31519507 PMC7015702

[ref25] Herman S. (2009). “Forensic child sexual abuse evaluations: accuracy, ethics, and admissibility” in The Evaluation of Child Sexual Abuse Allegations, a Comprehensive Guide to Assessment and Testimony. eds. Kuenhle K. Connell M. (Hoboken, NJ: Wiley), 247–266.

[ref26] Johnson M. Magnussen S. Thoresen C. Lønnum K. Burrell L. V. Melinder A. (2015). Best practice recommendations still fail to result in action: a national 10-year follow-up study of investigative interviews in CSA cases. Appl. Cogn. Psychol. 29, 661–668. doi: 10.1002/acp.3147

[ref27] Kask K. Pompedda F. Palu A. Schiff K. Mägi M.-L. Santtila P. (2022). Transfer of avatar training effects to investigative field interviews of children conducted by police officers. Front. Psychol. 13:753111. doi: 10.3389/fpsyg.2022.753111, PMID: 35874417 PMC9298842

[ref28] Khurana D. Koli A. Khatter K. Singh S. (2022). Natural language processing: state of the art, current trends and challenges. Multimed. Tools Appl. 82, 3713–3744. doi: 10.1007/s11042-022-13428-4, PMID: 35855771 PMC9281254

[ref29] Korkman J. Santtila P. Drzewiecki T. Kenneth Sandnabba N. (2008). Failing to keep it simple: language use in child sexual abuse interviews with 3–8-year-old children. Psychol. Crime Law 14, 41–60. doi: 10.1080/10683160701368438

[ref30] Korkman J. Santtila P. Sandnabba N. K. (2006). Dynamics of verbal interaction between interviewer and child in interviews with alleged victims of child sexual abuse. Scand. J. Psychol. 47, 109–119. doi: 10.1111/j.1467-9450.2006.00498.x, PMID: 16542353

[ref31] Krähenbühl S. Blades M. Eiser C. (2009). The effect of repeated questioning on children’s accuracy and consistency in eyewitness testimony. Leg. Criminol. Psychol. 14, 263–278. doi: 10.1348/135532508X398549

[ref32] Krause N. Pompedda F. Antfolk J. Zappalà A. Santtila P. (2017). The effects of feedback and reflection on the questioning style of untrained interviewers in simulated child sexual abuse interviews. Appl. Cogn. Psychol. 31, 187–198. doi: 10.1002/acp.3316

[ref33] Lamb M. E. Brown D. A. Hershkowitz I. Orbach Y. Esplin P. W. (2018). Tell me what happened: Questioning children about abuse. Hoboken, NJ: John Wiley & Sons.

[ref34] Lamb M. E. Hershkowitz I. Sternberg K. J. Esplin P. W. Hovav M. Manor T.. (1996). Effects of investigative utterance types on Israeli children’s responses. Int. J. Behav. Dev. 19, 627–637. doi: 10.1177/016502549601900310

[ref35] Lamb M. E. Sternberg K. J. Orbach Y. Esplin P. W. Mitchell S. (2002a). Is ongoing feedback necessary to maintain the quality of investigative interviews with allegedly abused children? Appl. Dev. Sci. 6, 35–41. doi: 10.1207/S1532480XADS0601_04

[ref36] Lamb M. E. Sternberg K. J. Orbach Y. Esplin P. W. Stewart H. Mitchell S. (2003). Age differences in young children’s responses to open-ended invitations in the course of forensic interviews. J. Consult. Clin. Psychol. 71, 926–934. doi: 10.1037/0022-006X.71.5.926, PMID: 14516241

[ref37] Lamb M. E. Sternberg K. J. Orbach Y. Hershkowitz I. Horowitz D. Esplin P. W. (2002b). The effects of intensive training and ongoing supervision on the quality of investigative interviews with alleged sex abuse victims. Appl. Dev. Sci. 6, 114–125. doi: 10.1207/S1532480XADS0603_2

[ref38] Landis J. R. Koch G. G. (1977). The measurement of observer agreement for categorical data. Biometrics 33, 159–174. doi: 10.2307/2529310 843571

[ref39] Lyon T. D. (2014). Interviewing children. Annu. Rev. Law Soc. Sci. 10, 73–89. doi: 10.1146/annurev-lawsocsci-110413-030913

[ref40] Malloy L. C. Orbach Y. Lamb M. E. Walker A. G. (2017). “How” and “why” prompts in forensic investigative interviews with preschool children. Appl. Dev. Sci. 21, 58–66. doi: 10.1080/10888691.2016.1158652

[ref41] McCambridge J. Witton J. Elbourne D. R. (2014). Systematic review of the Hawthorne effect: new concepts are needed to study research participation effects. J. Clin. Epidemiol. 67, 267–277. doi: 10.1016/j.jclinepi.2013.08.015, PMID: 24275499 PMC3969247

[ref42] Pompedda F. Antfolk J. Zappalà A. Santtila P. (2017). A combination of outcome and process feedback enhances performance in simulations of child sexual abuse interviews using avatars. Front. Psychol. 8:1474. doi: 10.3389/fpsyg.2017.01474, PMID: 28955259 PMC5601953

[ref43] Pompedda F. Palu A. Kask K. Schiff K. Soveri A. Antfolk J.. (2020). Transfer of simulated interview training effects into interviews with children exposed to a mock event. Nordic Psychol. 73, 43–67. doi: 10.1080/19012276.2020.1788417

[ref44] Pompedda F. Zappalà A. Santtila P. (2015). Simulations of child sexual abuse interviews using avatars paired with feedback improves interview quality. Psychol. Crime Law 21, 28–52. doi: 10.1080/1068316X.2014.915323

[ref45] Pompedda F. Zhang Y. Haginoya S. Santtila P. (2022). A mega-analysis of the effects of feedback on the quality of simulated child sexual abuse interviews with avatars. J. Police Crim. Psychol. 37, 485–498. doi: 10.1007/s11896-022-09509-7

[ref46] Powell M. B. Guadagno B. Benson M. (2016). Improving child investigative interviewer performance through computer-based learning activities. Polic. Soc. 26, 365–374. doi: 10.1080/10439463.2014.942850

[ref47] Salehi P. Hassan S. Z. Lammerse M. Sabet S. S. Riiser I. Røed R. K.. (2022). Synthesizing a talking child avatar to train interviewers working with maltreated children. Big Data Cogn. Comp. 6:62. doi: 10.3390/BDCC6020062

[ref48] Scullin M. H. Ceci S. J. (2001). A suggestibility scale for children. Personal. Individ. Differ. 30, 843–856. doi: 10.1016/S0191-8869(00)00077-5

[ref49] Shwartz-Ziv R. Armon A. (2022). Tabular data: deep learning is not all you need. Inform. Fusion 81, 84–90. doi: 10.1016/j.inffus.2021.11.011

[ref51] Sternberg K. J. Lamb M. E. Hershkowitz I. Esplin P. W. Redlich A. Sunshine N. (1996). The relation between investigative utterance types and the informativeness of child witnesses. J. Appl. Dev. Psychol. 17, 439–451. doi: 10.1016/S0193-3973(96)90036-2

[ref52] Sternberg K. J. Lamb M. E. Orbach Y. Esplin P. W. Mitchell S. (2001). Use of a structured investigative protocol enhances young children’s responses to free-recall prompts in the course of forensic interviews. J. Appl. Psychol. 86, 997–1005. doi: 10.1037/0021-9010.86.5.997, PMID: 11596815

[ref53] Tamm A. Krause N. Volbert R. (2022). “The ViContact VR training: virtual children and their response algorithm” in Annual Conference of the International Investigative Interviewing Research Group (IIIRG) (Winchester, England)

[ref54] Taylor P. J. Russ-Eft D. F. Chan D. W. L. L. (2005). A meta-analytic review of behavior modeling training. J. Appl. Psychol. 90, 692–709. doi: 10.1037/0021-9010.90.4.692 16060787

[ref55] Volpini L. Melis M. Petralia S. Rosenberg M. D. (2016). Measuring Children’s suggestibility in forensic interviews. J. Forensic Sci. 61, 104–108. doi: 10.1111/1556-4029.12987, PMID: 27404406

