# Supplementary material for: AI avatar tells you what happened: The first test of using AI-operated children in simulated interviews to train investigative interviewers
Source: Front Psychol. 2023 Feb 23;14:1133621. doi: 10.3389/fpsyg.2023.1133621 (PMC9995382; doi:10.3389/fpsyg.2023.1133621)
Supplement: Supplementary file 1 [file Data_Sheet_1.PDF]

Supplementary Material

AI Avatar Tells You What Happened: The First Test of Using AI-Operated Children in Simulated Interviews to Train Investigative Interviewers

Shumpei Haginoya[1*], Tatsuro Ibe[2], Shota Yamamoto[3], Naruyo Yoshimoto[4], Hazuki Mizushi[5], and Pekka Santtila[6]

*** Correspondence:** Shumpei Haginoya: haginoya@psy.meijigakuin.ac.jp

1 Class distributions of training and test data

Question types	Training Data	Test Data
Invitation broad	372	93
Invitation focus	303	76
Facilitator	1223	306
Directive	1812	453
Option posing and specific suggestive questions	1766	441
Unspecific suggestive	766	192
Inappropriate utterance for children	386	96

2 Instruction About Best Interview Practice

2.1 Guidelines for the correct questioning style

The behavior of children in interviews is considerably different from that of adults. Young children are especially vulnerable to suggestion: They tend to perceive any information provided by adults as true and do not always negate incorrect statements made by the interviewer. Because of that, there are established recommendations about question types to use when interviewing children: In general, open-ended question types are recommended. This is because they are most likely to elicit a reliable answer. Open-ended questions can be:

Invitations: Open-ended questions (questions, statements or imperatives) used to elicit free recall responses from the child. Invitations could be general ('Tell me everything that happened from the very beginning to the end') or relate to something just mentioned by the child ('Tell me more about that').

Facilitators: Non-suggestive encouragements to continue with a response. These include utterances like 'ok,' and restatements (echoing) of the child's previous utterance.

Directive questions: These refocus the child's attention on details already mentioned by the child and request further elaboration (for example, 'Where were you when that happened?').

On the other hand, closed and suggestive questions should be avoided, as the answers elicited by them are much less reliable and they might create false memories in children. Closed and suggestive questions include:

Option-posing questions: These focus the child's attention on issues that the child had not previously mentioned but do not imply that a particular response is expected. The answer to these types of questions is usually 'yes or no'. For example, the interviewer might ask 'Did he touch your penis?' or 'Did he do anything with his penis?'

Suggestive questions: These are stated in such a way that the interviewer strongly communicates what response was expected (for example: 'He forced you to do that, didn't he?'), or assumed details that had not been revealed by the child (for example: Child: 'We laid on the sofa.' Interviewer: 'He laid on you or you laid on him?').

2.2 Questions before the interview

1. If the child doesn't provide any detail regarding the alleged circumstances of abuse, the interviewer should ask the child questions related to the alleged situation. For example: Did your father touch you?

○ Yes

○ No

2. If the child provides a detail regarding the alleged events, for example 'he punched me' which is the best question to ask?

○ Did it hurt?

○ Who punched you?

○ Was it your father?

3 Background to the Interview

Haruna (4 years old)

Haruna is a 4-year-old girl. She is outgoing, although she has some developmental cognitive delays. She lives with her father, mother and brother Yota (12 years old). Occasionally her aunt also stays with them. She doesn't have an ideal family environment, due to the low educational level of her parents, and the fact that her mother (Narumi) is mentally disabled. Haruna does not go to kindergarten, since her father doesn't allow her to. Haruna's father (Tatsuya) is not her biological father, but he married Haruna's mother after Haruna's birth.

Yota takes care of their mother, along with the aunt, while his father takes care of Haruna. Yota and the aunt noticed some bruises on Haruna’s arms. They went to a doctor asking for a cure for those bruises, and the doctor did not exclude the possibility of abuse.
